# An Unsuspected Intraventricular Schwannoma

**DOI:** 10.7759/cureus.18683

**Published:** 2021-10-11

**Authors:** Chrystal Calderon, Amit Ramsingh, Devindra Ramnarine

**Affiliations:** 1 Neurosurgery, North Central Regional Health Authority, Champs Fleurs, TTO

**Keywords:** neurosurgical procedures, benign, lateral ventricles, schwannoma, intraventricular

## Abstract

Intraventricular schwannomas are rarely encountered in neurosurgical practice. The development and progression of a schwannoma within the ventricular system is still a hypothesised theory. Here, we present a case of a 59-year-old female who presented with a three-week history of headaches. Her symptoms progressively worsened, with resultant altered mental status within the last week. Imaging scans of the brain demonstrated a well-defined mass within the right lateral ventricle with associated midline shift and obstructive hydrocephalus. A parietal craniotomy and resection of the intraventricular mass was performed. Her postoperative course was uneventful. Histopathological assessment depicted a biphasic pattern of Antoni A and B, with a strongly positive S100. This was in keeping with an intraventricular schwannoma. The diagnosis of an intraventricular schwannoma does not fit within the classical differential framework for ventricular masses. These tumours are extremely uncommon but fortunately, they are typically benign. Therefore, adequate surgical resection remains the gold standard of care for these unfamiliar masses.

## Introduction

Schwannomas represent approximately 5%-8% of intracranial tumours [1]. These benign lesions typically arise from the nerve sheaths of peripheral and cranial nerves. The most common intracranial site is the vestibulocochlear nerve at the cerebellopontine angle [2,3]. Apart from its relation to cranial nerves, schwannomas are rare intracerebral tumours and account for less than 1% of intracranial schwannomas [1]. Schwannomas within the ventricular system are seldom noted and are usually a source of misdiagnosis on initial clinical assessment. Chiba et al reported under forty (40) published cases of this rare tumour in their paper [4]. The affected age group varies significantly for intracerebral schwannomas. Additionally, with the paucity of reported cases of intraventricular schwannomas in the literature, there is still more to be understood [3,4].

## Case presentation

A 59-year-old female, known hypertensive, presented to the emergency department with a three-week history of progressive generalized weakness and headaches. Within the last week, bowel and bladder incontinence was also noted. Her social history showed recreational use of cigarettes and marijuana. On presentation, she was in an acute confusional state with slurred speech. Her Glasgow coma scale (GCS) was assessed as 14 (fourteen) and there was normal power and tone in all limbs. However, she had appreciable left dysdiadochokinesia and past pointing. Cranial nerve function remained intact.

**Figure 1:**
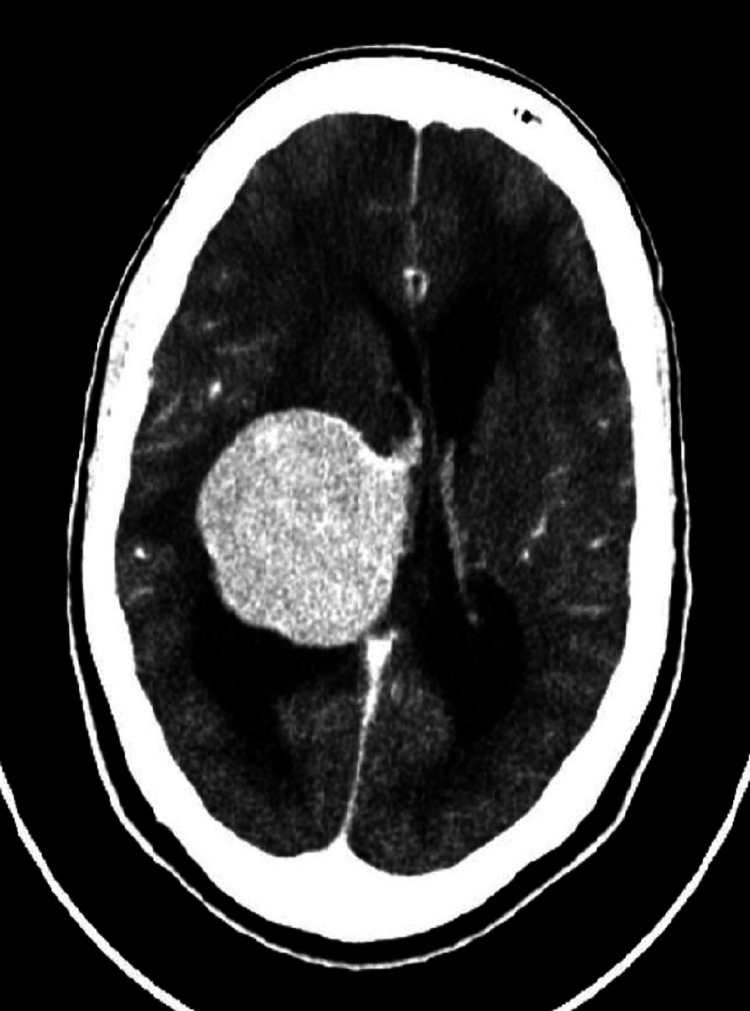
CT brain with contrast (axial view) demonstrating space-occupying lesion within the right lateral ventricle.

**Figure 2:**
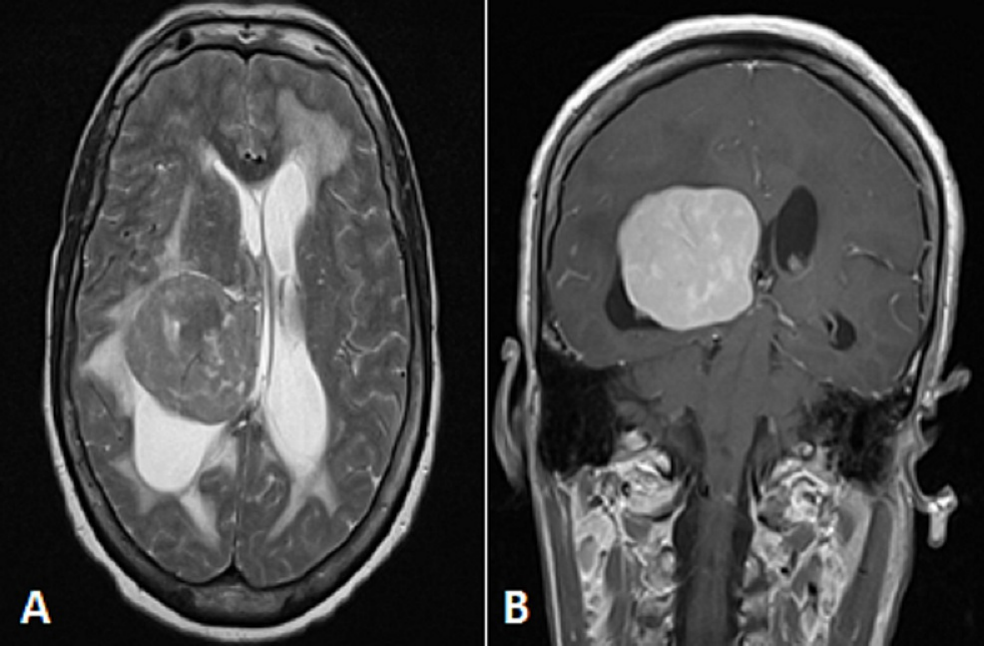
MRI brain with gadolinium. A: T2- weighted imaging (axial view) showing heterogenous isointense mass within the right lateral ventricle B: T1- weighted imaging (coronal view) showing contrast enhancement of tumour, with associated midline shift and obstructive hydrocephalus.

Computed tomography (CT) of the brain with contrast showed a 4.3cm x 4.6cm x 5.2cm well-defined mass arising from the posterior horn of the right lateral ventricle. This was associated with significant midline shift and effacement of the quadrigeminal cistern (Figure 1). Magnetic resonance imaging (MRI) with gadolinium revealed a heterogeneous mass within the posterior horn of the right lateral ventricle with accompanying perilesional oedema, and hydrocephalus. This mass was isointense on both T1-weighted and T2-weighted images (Figure 2). At this time, the preliminary diagnoses considered were a primary brain lesion such as a meningioma versus a secondary tumour. Staging CT declared that there was no evidence of suspicious lesions within the chest, abdomen and pelvis.

**Figure 3:**
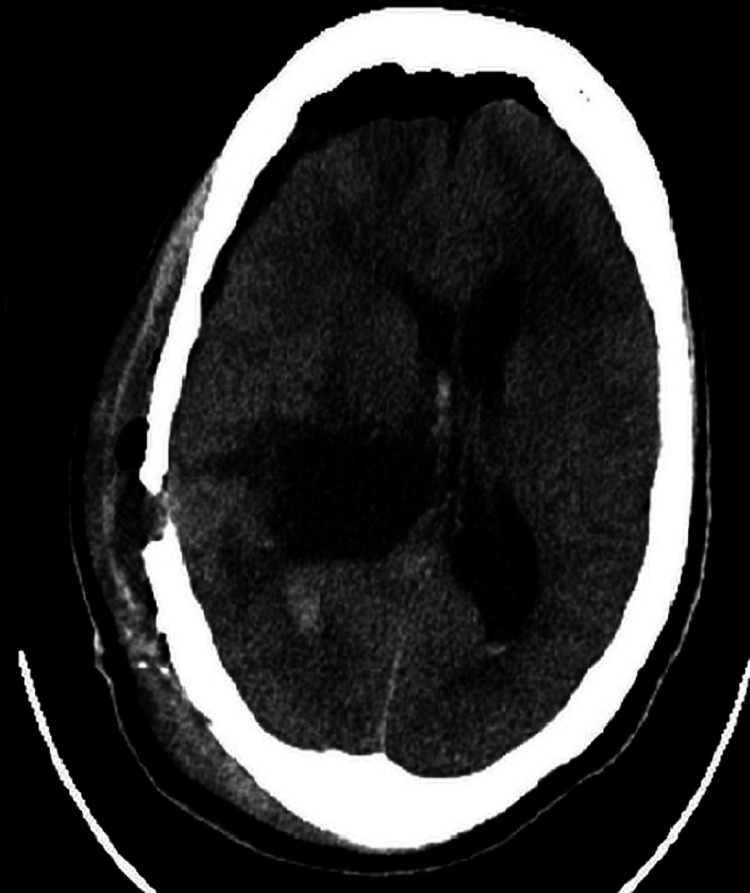
CT brain non-contrast demonstrating resection of tumour within the right lateral ventricle, with post-surgical changes.

**Figure 4:**
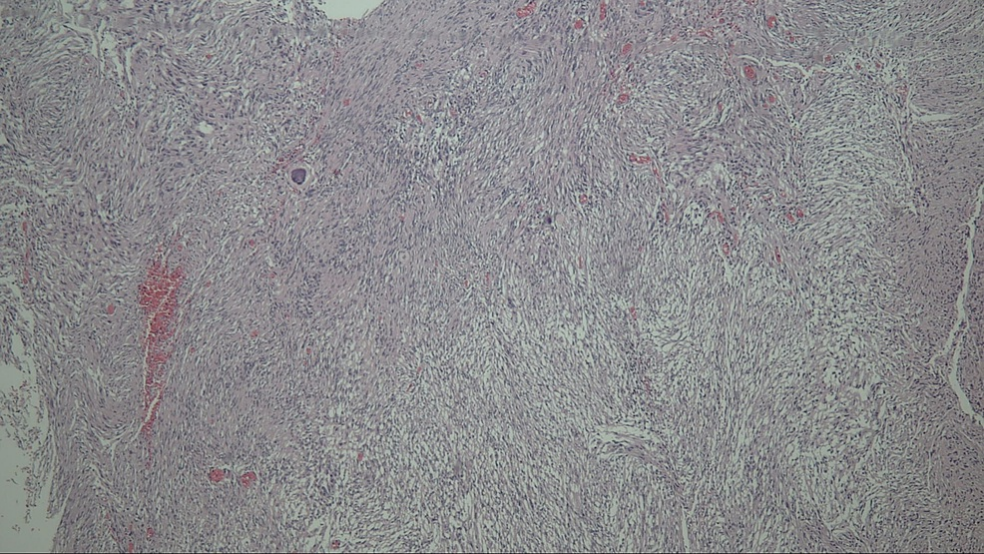
Histopathology showing bland spindle cells in biphasic pattern of hypocellular and hypercellular areas (H&E stained). H&E: hematoxylin and eosin.

High-dose intravenous corticosteroids were commenced, and tumour resection was scheduled urgently. A parietal craniotomy with trans-cortical access to the lateral ventricle was performed under general anaesthesia. Complete resection of the intraventricular tumour was achieved. The post-surgical course was uneventful, and her GCS returned to 15 (fifteen) on day one following surgery and thereafter. Her Karnofsky Performance Status score is 100% on follow-up. Repeat radiologic imaging demonstrated no gross evidence of residual tumour, with resolving hydrocephalus and midline shift (Figure 3). A CSF diversion procedure was not warranted in this case. Histopathological reporting of the resected mass indicated features of a cellular schwannoma with a biphasic cellular arrangement of Antoni A and B patterns. Additionally, a strong diffusely positive S100 and focal weak positive epithelial membrane antigen was depicted on immunohistochemistry (Figure 4).

## Discussion

Intraventricular schwannomas are exceedingly rare intracranial tumours [4]. When encountered, they are predominantly located within the lateral ventricles, followed by the fourth ventricle [5,6]. Given that these tumours typically arise from Schwann cells of peripheral and autonomic nerves, their appearance within the ventricular system is not explicable. It has been hypothesized the migration of ectopic debris of neural crest cells to the ventricles and the possible association of Schwann cells and the choroid plexus [6,7]. However, due to limited number of cases noted in the literature, the clinical course and aetiology is not ascertained.

Approximately 90% of these nerve sheath tumours occur sporadically, while the remainder is associated with familial tumour syndromes. Neurofibromatosis type 2 (NF 2) and the inactivation of the tumour suppressor gene, merlin, is one of the primary familial syndromes [8]. Interestingly, no correlation with familial syndromes and reported intraventricular schwannomas has been shown in the literature [3]. This was also depicted in this case, with no significant medical or family history of NF 2. The clinical features of these intracranial lesions are diverse and vary according to their site and size. Typical features include headaches, seizures and neurological deficits [1,9]. Intraventricular lesions are prone to causing features of obstructive hydrocephalus. Similarly, as described in this case report, this patient presented with an altered mental status and corresponding evidence of hydrocephalus on imaging.

Macroscopically, these lesions are usually well defined, which can displace surrounding structures without direct invasion. They may also show calcification, cystic degeneration, or haemorrhage. Microscopically, these tumours are described as biphasic, composed of spindle-shaped elongated cells with abundant eosinophilic cytoplasm. Hypercellular (Antoni A) and loose hypocellular (Antoni B) areas are noted [1,2]. Immunohistochemical analysis demonstrates strong positivity for S100 [1]. Generally, these tumours are usually slow-growing and infrequently undergo malignant transformation, but malignant intraventricular schwannomas have been reported [3].

Radiological imaging usually demonstrates a well-defined lesion, with or without evidence of cystic degeneration and calcification [1,9]. However, the differential diagnosis of an intraventricular mass alters based on the affected age group. Within the fifth to sixth decades of life, as seen here, more common aetiologies were considered. Such as possible meningioma, glioma, or metastatic lesion. Given the rare nature of intraventricular schwannomas, it is difficult to ascertain a diagnosis based solely on radiological assessment [5,10]. Fortunately, these tumours are typically benign, and a gross total surgical resection remains the mainstay. Long-term prognosis is overall good in this exceptional tumour group of cases [1].

## Conclusions

Neoplasms within the lateral ventricle in the adult neurosurgical patient are typically diagnosed as meningiomas, gliomas, choroid plexus tumours or possible metastasis. Intracerebral schwannomas, especially intraventricular schwannomas, are extremely uncommon. Based simply on radiological assessment, the rate of misdiagnosis will be high. This is case demonstrated the extraordinary diagnosis of an intraventricular schwannoma in the lateral ventricle of a middle-aged female, which will add to the limited published data available. Although this tumour is infrequently encountered in clinical practice, a clear histopathological diagnosis with or without immunohistochemistry is essential. These tumours are usually benign, and a complete surgical resection will result in an overall good prognostic outcome, as in this case.
